# Multimodality therapy for stage IB3 cervical cancer with a delayed diagnosis at 21 weeks of gestation: from neoadjuvant chemotherapy to adjuvant chemoradiotherapy—a case report

**DOI:** 10.3389/fmed.2026.1946977

**Published:** 2026-09-23

**Authors:** Xinjun Li, Shuo Xu, Yanan Ren, Mei Li, Xiangnan Zhang, Ren Xu, Shihao Liu, Shengpu Wang

**Affiliations:** 1 Department of Gynecology, Hebei General Hospital, Shijiazhuang, China; 2 Department of Pathology, Hebei General Hospital, Shijiazhuang, China; 3 Department of Gynecology, The Fourth Hospital of Hebei Medical University, Shijiazhuang, China; 4 Department of Obstetrics, The Second Hospital of Hebei Medical University, Shijiazhuang, China

**Keywords:** case report, cervical cancer, neoadjuvant chemotherapy, pregnancy, radical hysterectomy, SCC antigen, stage IB3

## Abstract

**Background:**

Gestational cervical cancer is rare, with an incidence of 1.4–4.6 per 100,000 pregnancies. Managing locally advanced cervical cancer in the second trimester is challenging, requiring a balance between maternal oncological safety and fetal viability. No standardized protocol exists for stage IB3 cervical cancer diagnosed at 21 weeks of gestation.

**Case presentation:**

A 27-year-old primigravida at 21 weeks of gestation was diagnosed with stage IB3 cervical squamous cell carcinoma. Initial SCC was 60.15 ng/mL, and MRI was suspicious for stage IIB disease. The patient had no routine prenatal care, and placenta previa on ultrasound contributed to misdiagnosis and delayed recognition. Following multidisciplinary team consultation, she received four cycles of neoadjuvant chemotherapy (nab-paclitaxel 400 mg + cisplatin 140 mg per cycle). Serial SCC showed initial response, a plateau with mild elevation after the third cycle, and a modest decline after the fourth (29.33 ng/mL). At 36 weeks, after dexamethasone for fetal lung maturation, concurrent cesarean section and radical surgery (hysterectomy, lymphadenectomy, ovarian transposition) were performed. A live female neonate (2095g, Apgar 10/10) was delivered. Pathology confirmed moderately differentiated squamous cell carcinoma with intermediate stromal invasion (>1/3 and <2/3) and perineural invasion, without lymphovascular invasion or nodal metastasis. Adjuvant pelvic radiotherapy with concurrent cisplatin and vaginal brachytherapy was given. At last follow-up (June 2026), SCC remained normal with no recurrence, and the child showed normal development.

**Conclusion:**

Sequential multimodal therapy—NACT, delayed radical surgery at 36 weeks, and adjuvant chemoradiotherapy—is feasible for stage IB3 cervical cancer in the second trimester. Serial SCC monitoring is a useful biomarker for guiding treatment decisions. This case highlights pitfalls including diagnostic delay, radiological overstaging, and false-negative TCT, emphasizing the need for thorough examination and high suspicion in pregnant patients with atypical bleeding.

## 1 Introduction

Cervical cancer represents one of the most common malignant tumors complicating pregnancy, with a reported incidence of 1.4–4.6 per 100,000 pregnancies (1). In recent years, delayed childbearing and the younger onset of cervical malignancy have increased the overlapping prevalence of pregnancy and cervical cancer. However, the clinical management of locally advanced cervical cancer diagnosed in the second trimester remains controversial, and standardized treatment guidelines for gestational stage IB3 cervical cancer are currently unavailable (2).

Current clinical strategies depend on comprehensive evaluation of pathological features, clinical tumor staging, fetal gestational maturity, and maternal fertility intention. For stage IB3 cervical cancer (tumor diameter >4 cm) diagnosed after 14 weeks of gestation, platinum-based NACT is the primary conservative approach to inhibit tumor progression while allowing sufficient time for fetal maturation (3). Nevertheless, the optimal timing of delivery and definitive radical surgery, the necessity and regimen of adjuvant therapy, and long-term maternal and neonatal prognostic outcomes require further clinical validation.

Herein, we report a rare case of stage IB3 cervical squamous cell carcinoma with a delayed diagnosis at 21 weeks of gestation, managed with a standardized multimodal sequential treatment strategy including NACT, delayed combined cesarean-radical surgery at 36 weeks, and risk-adapted adjuvant chemoradiotherapy. We focus on dynamic SCC biomarker-guided treatment adjustment, MDT-based individualized decision-making, and 4-year long-term follow-up outcomes, aiming to supplement clinical evidence for the standardized management of gestational locally advanced cervical cancer.

## 2 Case report

### 2.1 Patient information

A 27-year-old primigravida presented to Hebei General Hospital on June 19, 2022, with a 3-month history of irregular vaginal bleeding at 21 + 2 weeks of gestation. The patient had no prior regular prenatal examination. Early gestational ultrasound indicated placenta previa, and the vaginal bleeding was initially attributed to placental abnormality. Conservative management with bed rest and oral progesterone was administered, yet bleeding symptoms persisted without improvement.

### 2.2 Diagnostic workup

Pelvic examination revealed loss of normal cervical architecture with an approximately 8 × 6 cm cauliflower-like exophytic mass that was firm and bled easily on contact. Rectovaginal examination showed an enlarged cervix (approximately 8 cm in diameter) with soft parametrial tissues and no palpable nodules.

Cervical cytology (Thinprep Cytologic Test, TCT) showed no intraepithelial lesions or malignant cells, but high-risk human papillomavirus (HR-HPV) testing was positive for HPV 16. The baseline serum SCC level was markedly elevated at 60.15 ng/mL on June 20, 2022.

Colposcopy-guided cervical biopsy was performed on June 21, 2022, which confirmed moderately differentiated squamous cell carcinoma of the cervix. Pelvic MRI conducted on June 25, 2022, demonstrated a bulky cervical mass measuring approximately 70 × 38 × 64 mm and was suspicious for stage IIB disease (Figure 2A). Systemic imaging evaluation excluded distant metastasis.

Combining clinical manifestations, imaging features, and pathological results, the final clinical diagnosis was gestational cervical squamous cell carcinoma, stage IB3, at 21 weeks of gestation.

### 2.3 Therapeutic intervention

Given the patient's strong desire to continue the pregnancy, a multidisciplinary team (MDT) conference was conducted. The team comprised specialists from gynecologic oncology, obstetrics, medical oncology, reproductive genetics, neonatology, and radiology, who collaboratively formulated an individualized treatment protocol.

The MDT faced a critical dilemma: initiating chemotherapy could potentially jeopardize fetal safety, while delaying definitive treatment until fetal maturity risked maternal oncological progression. After extensive deliberation, the consensus strategy was defined as: preoperative NACT with regular fetal ultrasound monitoring, followed by delayed combined cesarean section and radical surgery after fetal lung maturation.

The patient received four consecutive cycles of NACT with nab-paclitaxel (400 mg) plus cisplatin (140 mg) at gestational ages of 22 + 3, 25 + 5, 28 + 5, and 32 + 5 weeks, respectively. Based on the patient's body surface area of 1.56 m^2^, this flat dosing corresponded to approximately 256 mg/m^2^ for nab-paclitaxel and 90 mg/m^2^ for cisplatin per cycle. The nab-paclitaxel dose was consistent with the standard neoadjuvant regimen for cervical cancer (260 mg/m^2^). The cisplatin dose of 90 mg/m^2^ fell within the high-dose range (80–120 mg/m^2^) as defined in the cisplatin package insert, which was selected by the MDT based on the patient's good performance status and favorable organ function, with the aim of maximizing the cytoreductive effect prior to definitive surgery. The patient tolerated the regimen well without dose-limiting toxicity. The choice of nab-paclitaxel over conventional paclitaxel was based on its albumin-bound formulation, which eliminates the need for corticosteroid and antihistamine premedication, reduces solvent-related toxicity, and may offer more predictable tumor delivery—advantages particularly relevant in the gestational setting where minimizing unnecessary medication exposure is desirable. The choice of cisplatin over carboplatin was based on its established efficacy in neoadjuvant and concurrent chemoradiotherapy settings for cervical cancer. Serial dynamic SCC levels were recorded as follows: baseline 60.15 ng/mL; 46.36 ng/mL (22.9% reduction) after the first cycle; 34.74 ng/mL (25.1% reduction) after the second cycle; 37.48 ng/mL (7.9% elevation) after the third cycle; and 29.33 ng/mL (21.7% reduction) after the fourth cycle (Figure 1). The interval between the third and fourth cycles was extended to 4 weeks instead of the scheduled 3 weeks due to the patient experiencing grade 2 neutropenia, which required an additional week for hematologic recovery. Pelvic MRI reexamination confirmed significant shrinkage of the cervical mass after sequential chemotherapy, consistent with biochemical response trends (Figure 2B).

**Figure 1:**
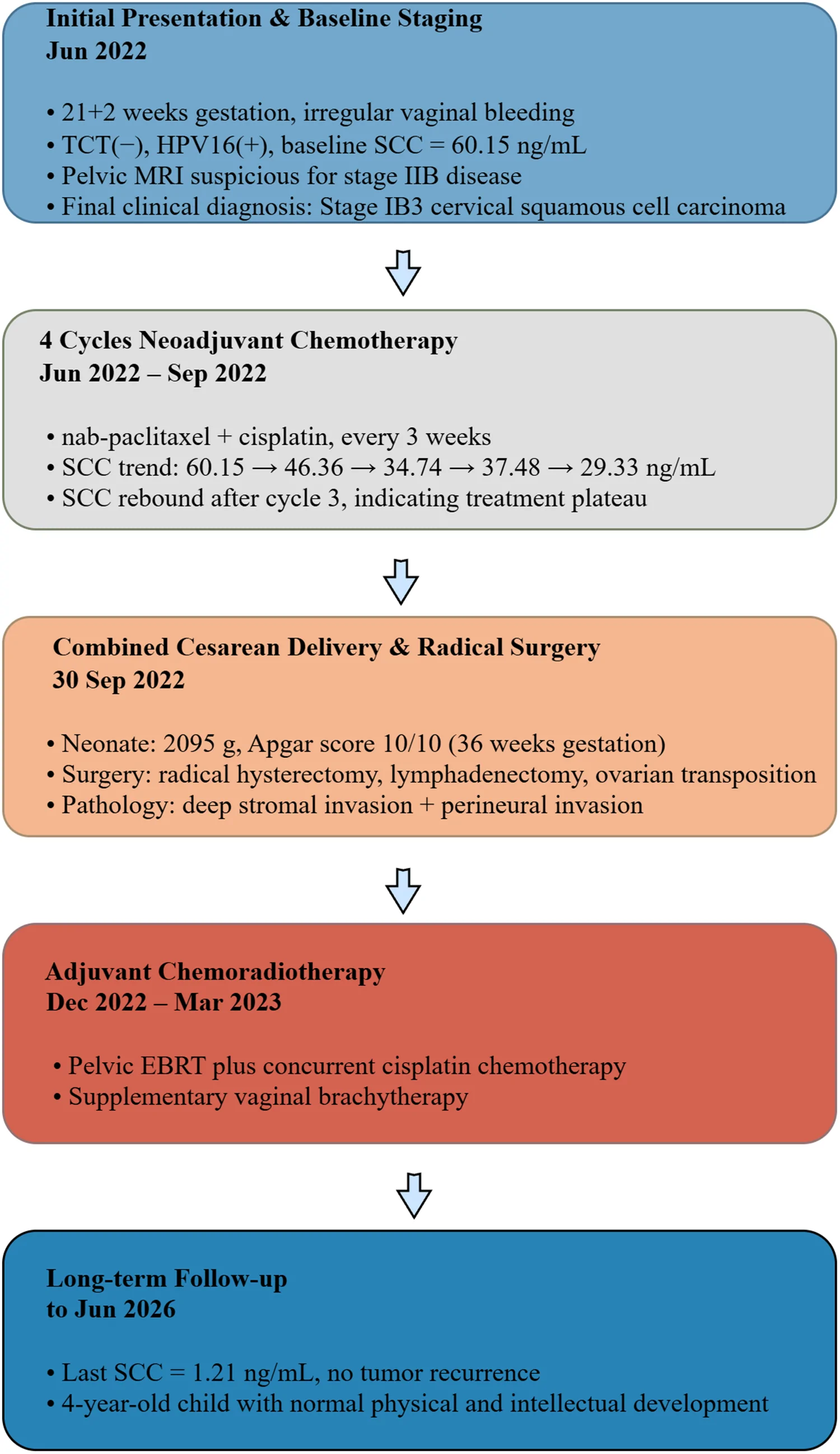
Treatment timeline and SCC dynamics. Clinical timeline outlining the sequence of initial presentation, neoadjuvant chemotherapy, combined cesarean delivery and radical surgery, adjuvant chemoradiotherapy, and long-term follow-up, with serial SCC levels shown throughout the entire treatment course.

**Figure 2:**
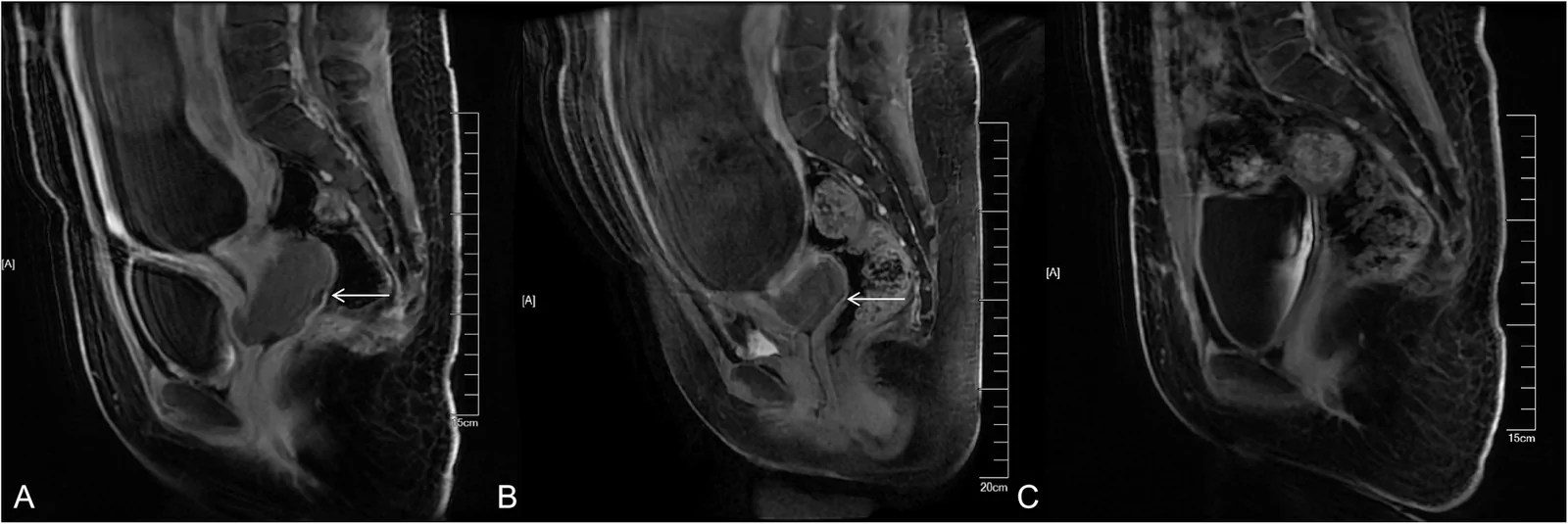
Longitudinal comparison of pelvic MRI images. Serial contrast-enhanced T1-weighted MRI images at three key time points: **(A)** initial diagnosis (June 2022) showing a bulky cervical tumor with suspected parametrial invasion (arrow), measuring approximately 70 × 38 × 64 mm; **(B)** post-NACT (September 2022) showing significant tumor shrinkage, measuring approximately 49 × 30 × 64 mm; **(C)** post-adjuvant therapy (January 2023) showing complete elimination of residual lesions without recurrence.

Given the biochemical plateau indicating limited further benefit from continued chemotherapy, and the achievement of fetal lung maturity at 36 weeks of gestation, a multidisciplinary decision was made to proceed with delivery and definitive surgery. Intramuscular dexamethasone was administered to promote fetal lung maturation prior to the procedure. A multidisciplinary collaborative operation was performed on September 30, 2022. A live female neonate weighing 2095g with a 1-minute Apgar score of 10/10 was delivered via lower segment cesarean section and transferred to the neonatal intensive care unit (NICU) for routine observation. Concurrent radical oncological surgery included type III radical hysterectomy, bilateral salpingectomy, pelvic and para-aortic lymphadenectomy, and intraoperative bilateral ovarian transposition to the paracolic sulci at the iliac crest level to preserve ovarian endocrine function.

### 2.4 Postoperative pathological findings

Postoperative pathological examination confirmed moderately differentiated squamous cell carcinoma with cervical wall stromal invasion depth between 1/3 and 2/3, accompanied by perineural invasion (Figure 3B). The tumor measured approximately 4 × 5 × 6 cm on pathological examination of the surgical specimen. Pathological assessment demonstrated a partial response to neoadjuvant chemotherapy, with a tumor regression grade of 2 (CRS 2), characterized by partial regression with fibrotic changes, though residual viable tumor remained readily identifiable. No lymphovascular invasion or tumor thrombus was observed, and all surgical resection margins were tumor-negative. Immunohistochemical staining confirmed the squamous phenotype of the tumor, with diffuse positivity for P16, P40, and P63, scattered P53 positivity, and a Ki-67 proliferation index of approximately 60%. A total of 15 regional lymph nodes were examined, and no pelvic or para-aortic lymph node metastasis was identified. Additional placental pathological findings included villous adhesion, capillary congestion, multifocal fibrinoid deposition, and focal calcification. The postoperative serum SCC level normalized to 1.35 ng/mL within the normal reference range on December 26, 2022 (Figure 1).

**Figure 3:**
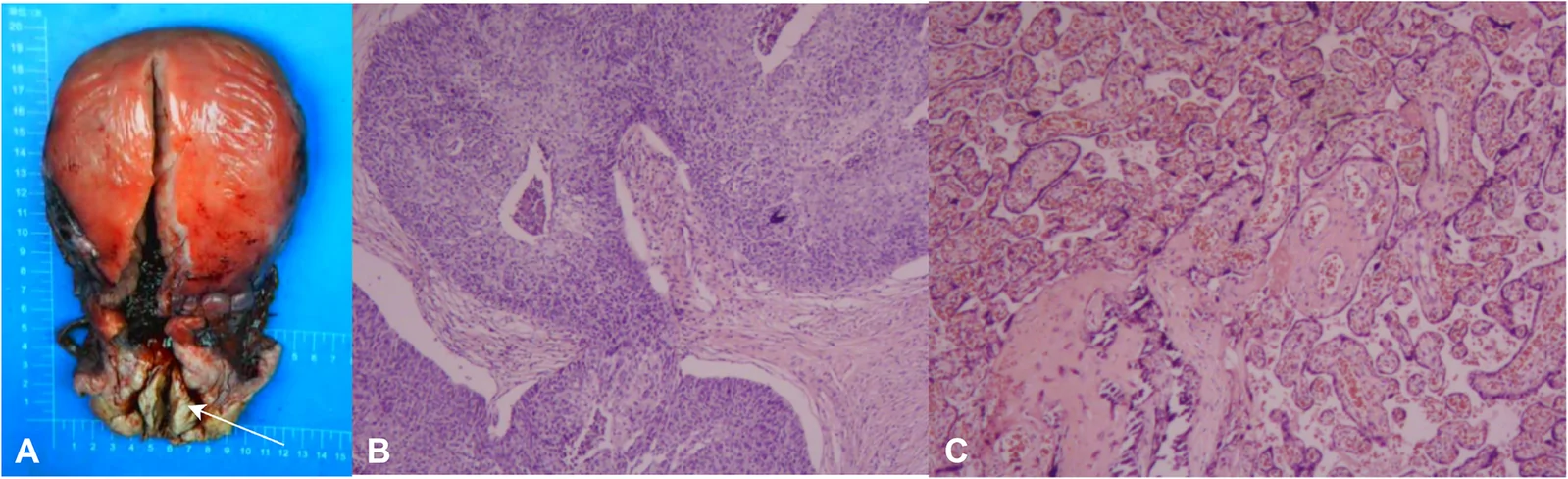
Gross and histopathological findings. **(A)** Gross specimen of the uterus and cervical tumor following neoadjuvant chemotherapy, with the arrow indicating the cervical lesion. **(B)** Hematoxylin-eosin (H&E) staining of cervical squamous cell carcinoma, original magnification ×200. **(C)** H&E staining of placental tissue, original magnification ×200, showing villous congestion, fibrinoid deposition, and focal calcification.

### 2.5 Adjuvant therapy

According to the Sedlis criteria, for patients with negative lymphovascular space invasion, the presence of middle- or deep-third stromal invasion and a tumor size ≥4 cm fulfills the intermediate-risk criteria. Given the patient's intermediate-risk factors (middle-third stromal invasion and initial tumor size of 8 cm) meeting the Sedlis criteria, combined with the adverse prognostic factor of perineural invasion, adjuvant pelvic external beam radiotherapy was initiated on December 30, 2022. A total dose of 45 Gy was delivered in 23 fractions (1.96 Gy per fraction) to the pelvic field, including the common iliac, internal iliac, external iliac, and obturator lymphatic drainage areas, as well as the region surrounding the vaginal stump extending to the upper half of the vagina. After completion of external beam radiotherapy, high-dose-rate vaginal brachytherapy was administered as a boost, with a prescribed dose of 6 Gy per fraction for 3 fractions, prescribed to the vaginal mucosal surface, covering the upper 3 cm of the vagina. Concurrent low-dose cisplatin chemotherapy (30 mg on days 1–2 per cycle) was administered for four cycles from January to February 2023.

The interval between surgery (September 30, 2022) and initiation of radiotherapy (December 30, 2022) included a recovery period following radical surgery and intensive neonatal care. This timing also reflected the patient's preference for uninterrupted neonatal bonding during the immediate postpartum period, which was respected as part of the shared decision-making process.

### 2.6 Follow-up and prognostic outcomes

Long-term follow-up was maintained until June 2026, with the last follow-up on June 18, 2026. The patient remained disease-free with persistent normal serum SCC levels (with the last SCC level at 1.21 ng/mL) and negative systemic imaging examinations, without evidence of tumor recurrence or distant metastasis. Despite preterm birth and low birth weight, the neonate received standardized NICU care and exhibited completely normal physical growth and intellectual development at the 4-year follow-up, consistent with age-matched healthy children. The child's development was assessed using the Ages and Stages Questionnaire (ASQ) at well-child visits, with age-appropriate performance across all domains (gross motor, fine motor, communication, problem-solving, and personal-social skills).

## 3 Discussion

We report a well-documented case of stage IB3 cervical cancer with a delayed diagnosis at 21 weeks of gestation, successfully treated with sequential NACT, delayed radical surgery, and risk-stratified adjuvant chemoradiotherapy, with favorable 4-year maternal and neonatal prognostic outcomes. Five key clinical insights and one study limitation are summarized and discussed below.

### 3.1 Diagnostic delay from misattribution to placenta previa

The patient experienced a diagnostic delay due to the initial misattribution of vaginal bleeding to placenta previa. The absence of routine prenatal screening and inconclusive ultrasound findings obscured the underlying cervical malignancy. Clinically, gestational vaginal bleeding is preferentially attributed to obstetric etiologies, and clinicians often hesitate to perform speculum examination and colposcopy during pregnancy due to gestational safety concerns, further delaying the diagnosis of gestational cervical cancer (4). This case reinforces that placental lesions do not exclude concurrent cervical malignancy. For pregnant patients with persistent irregular vaginal bleeding, routine cervical speculum examination is mandatory to rule out malignant lesions, rather than empirically attributing symptoms to obstetric factors. Lack of prenatal cervical screening is an independent risk factor for delayed diagnosis of gestational cervical cancer.

### 3.2 False-negative TCT: an inherent diagnostic pitfall in gestational cervical cancer

Notably, the patient presented with an 8 cm bulky exophytic cervical tumor, whereas the preoperative TCT result was completely negative, fully reflecting the inherent limitations of cervical cytological screening in pregnant populations. The false-negative rate of cervical cytology screening ranges from 2% to 50% in different clinical populations (5). Physiological gestational changes, including cervical congestion, hormone-induced reactive cellular changes, excessive cervical mucus secretion, and incomplete exposure of the cervical transformation zone, significantly reduce the sampling accuracy of abnormal cells and contribute to false-negative cytological results (6). Retrospective data have confirmed that pregnant patients with cervical cancer and negative initial cytology results have significantly longer diagnostic intervals and are more likely to be diagnosed at advanced tumor stages (5). Consistent with current ASCCP guidelines, HPV 16 positivity prompted timely colposcopic biopsy and corrected the misleading TCT result (7). This case strongly demonstrates that HPV genotyping and clinical pelvic examination are more reliable than isolated TCT cytology for high-risk screening of gestational cervical lesions, and biopsy verification should be performed for clinically suspected cervical masses regardless of negative cytological findings.

### 3.3 Staging discordance: MRI overstaging vs. clinical and pathological staging

Preoperative pelvic MRI was suspicious for parametrial invasion (stage IIB), while systematic preoperative rectovaginal palpation revealed no parametrial nodules or sclerosis, leading to a clinical stage IB3 diagnosis. Postoperative pathological examination finally confirmed negative parametrial tissues without invasive lesions. The staging discordance between imaging and clinical pathology is multifactorial. On the one hand, gestational physiological changes, including pelvic hypervascularity, cervical edema, and pelvic ligamentous laxity, easily mimic parametrial invasive signs on MRI, resulting in imaging overstaging (8). On the other hand, NACT may have eradicated microscopic parametrial lesions that could not be detected by clinical examination or imaging before treatment.

Existing literature reports that the overall consistency between MRI and clinical staging of cervical cancer is only approximately 63%, with a considerable false-positive rate in parametrial invasion evaluation (9). For pregnant patients with suspicious MRI parametrial invasion but negative clinical examination findings, imaging results should not be the sole basis for abandoning surgical treatment. The MDT strategy of administering NACT to stabilize tumor progression and delaying radical surgery until fetal maturity effectively avoids overtreatment while preserving the opportunity for radical resection. Negative surgical margins and long-term disease-free survival provide supporting evidence for the feasibility and safety of this individualized staging and treatment pathway.

### 3.4 SCC dynamics guide chemotherapy termination and surgical timing

Serial SCC monitoring provided objective, real-time biomarkers for tumor response assessment and individualized treatment decision-making throughout therapy. The tumor presented obvious chemosensitivity in the first two NACT cycles, with the SCC level declining steadily from 60.15 ng/mL to 34.74 ng/mL. After a third cycle, the rate of decline slowed, with SCC level changing from 34.74 ng/mL to 37.48 ng/mL. A fourth cycle was administered, after which the SCC level decreased to 29.33 ng/mL. The total tumor marker reduction rate from baseline reached only 51%, remaining below the 60% threshold predictive of a favorable pathological response as reported in previous studies (10). Crucially, even after four cycles, the SCC value remained markedly elevated at approximately 20 times the upper normal limit, suggesting that continued chemotherapy was unlikely to achieve a complete response.

Notably, although the SCC reduction after four cycles (51%) fell short of the 60% threshold, the final pathological assessment demonstrated a partial response (tumor regression grade CRS 2) with negative margins and no nodal metastasis. This apparent discordance may reflect that the 60% threshold was derived from non-pregnant populations, and pregnancy-associated physiological changes—including increased plasma volume and altered drug distribution—may affect SCC clearance kinetics independently of true tumor response. This observation underscores that SCC dynamics should be interpreted as one component of a multimodal assessment, integrated with imaging and clinical findings.

In this case, dynamic SCC monitoring provided supportive information for tumor response assessment, complementing imaging findings and clinical evaluation. While MRI findings were confounded by gestational physiological changes, SCC trends offered additional data points that informed the MDT's decision-making (11). We acknowledge that this observation is derived from a single case and should not be interpreted as establishing SCC as a definitive surrogate marker for imaging, nor as a standalone criterion for treatment decisions in this population.

The MDT's decision to terminate chemotherapy at 36 weeks was based on a combination of factors rather than SCC trajectory alone. First, at 36 weeks of gestation, fetal lung maturity had been achieved, and the neonatal benefits of reaching this milestone were well established. Second, although the SCC level continued to decline after the fourth cycle, the rate of reduction had markedly slowed (21.7% after cycle 4, compared with 22.9% and 25.1% after cycles 1 and 2). Third, the absolute SCC value remained markedly elevated at approximately 20 times the upper normal limit. Given these factors—fetal maturity, the slowing rate of biochemical response, and the persistently high SCC level—the MDT decided that the risks of further delaying definitive surgery outweighed the potential benefits of continued chemotherapy. This decision achieved an optimal balance between oncological control and fetal safety: late preterm delivery at 36 weeks significantly reduces neonatal mortality and morbidity compared with earlier gestational termination (12), while timely radical surgery avoids further tumor progression. The favorable 4-year disease-free survival supports the soundness of this decision, though we acknowledge that it was individualized to this patient's circumstances.

### 3.5 Multimodal therapy rationale and long-term prognostic value

The one-stage combination of cesarean section and radical hysterectomy avoids the physical trauma and tumor progression risk of secondary surgery, and enables immediate postoperative pathological risk stratification to guide subsequent adjuvant therapy. Intraoperative bilateral ovarian transposition effectively preserves maternal ovarian endocrine function and prevents irreversible ovarian failure caused by pelvic radiotherapy in young patients (13).

Perineural invasion (PNI) has been reported as a potential adverse prognostic factor in cervical cancer. Some studies have shown that PNI may be associated with established intermediate-risk factors, including tumor size, deep stromal invasion, and lymphovascular space invasion, as well as with high-risk factors such as lymph node metastasis (14, 15). In addition, PNI has been reported to be associated with overall survival and disease-free survival in early-stage cervical cancer in several studies (16, 17). A review has also suggested that PNI status might be considered in the decision-making process for adjuvant therapy (18). In one published series, the presence of PNI was correlated with conventional indications for adjuvant therapy (odds ratio 12.0, *P* = 0.003), and 92% of PNI-positive patients in that series received postoperative radiation therapy or concurrent chemoradiotherapy (14).

In the present case, the patient already met the Sedlis criteria based on intermediate-risk factors (middle-third stromal invasion and initial tumor size of 8 cm). The presence of PNI was considered by the MDT as an additional factor that might increase the cumulative recurrence risk. After thorough discussion with the patient, adjuvant concurrent chemoradiotherapy was administered to optimize local control and reduce recurrence risk. The patient tolerated the regimen well without dose-limiting toxicity and achieved 4-year disease-free survival, suggesting that this individualized treatment approach was feasible in this case.

This case adds to the limited literature on multimodal management of stage IB cervical cancer during pregnancy, with a treatment trajectory similar to that reported in a recent stage IB2 case (19). The patient completed all adjuvant treatments smoothly without severe adverse reactions and achieved 4-year sustained disease-free survival. For the neonate, accumulating clinical evidence confirms that platinum-based chemotherapy administered after the first trimester does not increase the risks of congenital malformation, physical dysplasia, or neurodevelopmental impairment in offspring (20). The normal physical and intellectual development of the child at 4-year follow-up further supports the safety of second-trimester platinum-based NACT for fetal prognosis.

### 3.6 Study limitations

This study is a single-center, single-case report with inherent limitations. The treatment strategy was formulated based on individual patient conditions, MDT consensus, and the patient's fertility intention, which may not be directly generalizable to all patients with gestational stage IB3 cervical cancer. In addition, although 4-year follow-up confirmed favorable maternal and neonatal outcomes, longer-term follow-up is still required to verify the durable oncological prognosis and long-term developmental safety of the offspring. Furthermore, as a single case report, it is not possible to disentangle the specific treatment effect from the inherent tumor biology, and the favorable outcomes observed may reflect both therapeutic intervention and favorable disease characteristics.

## 4 Patient's perspective

The patient reported significant psychological distress after being diagnosed with cervical cancer at 21 weeks of gestation, mainly stemming from dual concerns regarding maternal tumor prognosis and fetal developmental safety. She was particularly anxious about the potential adverse effects of prenatal chemotherapy on the fetus. Professional MDT counseling on the safety and standardized application of second-trimester platinum-based chemotherapy effectively relieved her psychological anxiety and strengthened her confidence in completing systematic treatment and continuing pregnancy.

During the NACT cycle, the patient only experienced mild, clinically manageable fatigue and nausea without severe chemotherapy-related adverse events. She highly recognized the value of multidisciplinary collaborative management, which enabled her to successfully complete four cycles of standardized chemotherapy and safely prolong pregnancy to 36 weeks of gestation.

At the 4-year follow-up, the patient had completely recovered without any long-term treatment-related complications, and her quality of life returned to the normal level. The healthy physical and intellectual development of her daughter provided her with great psychological reassurance. She voluntarily consented to share her diagnosis and treatment experience, aiming to provide practical reference and emotional encouragement for other pregnant women facing gestational cervical cancer diagnosis.

## Data Availability

The original contributions presented in the study are included in the article/Supplementary Material, further inquiries can be directed to the corresponding author.
